# Ketamine alleviating depressive-like behaviors is associated with regulation of nNOS–CAPON–Dexras1 complex in chronic unpredictable mild stress rats

**DOI:** 10.1515/tnsci-2022-0245

**Published:** 2022-09-21

**Authors:** Yiwei Shen, Feng Lv, Su Min, Xuechao Hao, Jian Yu

**Affiliations:** Department of Anesthesiology, The First Affiliated Hospital of Chongqing Medical University, No. 1 Youyi Rd, Chongqing 400016, People’s Republic of China

**Keywords:** ketamine, depression, neuronal nitric oxide synthase, CAPON protein, Dexras1 protein

## Abstract

**Background:**

A growing number of studies have demonstrated that ketamine induces rapid and sustained antidepressant action. Neuronal nitric oxide synthase (nNOS) signaling has been explored for the treatment of neuropsychiatric disorders for decades. But the effect of ketamine on nNOS signaling is poorly understood. The aim of the present study was to investigate the effect of ketamine on nNOS signaling in a chronic unpredictable mild stress (CUMS) model of depression.

**Methods:**

Forty-eight rats were randomly divided into four groups: the control group of healthy rats (group C), the healthy rats treated with ketamine 10 mg/kg for 3 days (group CK), the rats model of stress-induced depression group (group D), and the depressed group treated with ketamine 10 mg/kg for 3 days (group DK). The sucrose preference test and open field test were used to assess behavioral changes. Immunohistochemistry, immunofluorescence, and real-time PCR analysis were carried out to measure the expression of nNOS, CAPON, and Dexras1 in the prefrontal cortex (PFC) of the CUMS rats.

**Results:**

Compared with healthy rats, the total distance traveled, the rearing counts, the sucrose preference percentage (SPP), and CAPON and Dexras1 expression in the PFC significantly decreased, while nNOS expression increased in CUMS rats. After treating with ketamine, the total distance traveled, the rearing counts, the SPP, and CAPON and Dexras1 expression significantly increased, while nNOS expression significantly decreased.

**Conclusion:**

The results indicated that ketamine improved the depressive behavior of rats, which may be related to the reduced nNOS expression and enhanced CAPON and Dexras1 expression.

## 1 Introduction

Major depressive disorder is a devastating mental disorder affecting ∼16% of the world population, causing serious health and socioeconomic consequences [1]. Although interventions such as antidepressants are available, a high proportion of patients remain treatment resistant [2]. Furthermore, the classical antidepressant drugs, which is predominantly monoamine-based, were found to have some shortcomings, these antidepressants have their own drawbacks, such as a lag period of 2–4 weeks before these molecules start providing relief to this depressed population [3]. Therefore, discovering and developing new antidepressant drugs are of great importance for pharmacotherapy of depression.

Recently, previous clinical studies and animal experiments have shown that ketamine, a *N*-methyl-d-aspartate (NMDA) receptor antagonist, has a rapid and sustained antidepressant-like effect, and it is now regarded as a potential therapeutic target for the treatment of major depression [4–8], as esketamine was approved by the FDA in 2019 for treatment [9]. However, the underlying mechanisms of the drug are still unclear so far.

Nitric oxide (NO) signaling, as a well-documented excitatory neurotransmitter signaling within the brain, has been shown to play an important role in brain function and has been proposed to be involved in the pathophysiology of depression and the mechanism underlying antidepressant action [10–12]. In the brain, the activation of NMDA receptors can promote the activation of nitric oxide synthase (NOS), which leads to the production of NO from l-amino acids, and NO in turn enhances glutamate release from pre-synaptic neurons [13]. Previous studies have reported that neuronal NOS (nNOS), the major NOS in the brain, is significantly increased in the brain of depressed animals exposed to stress [14–16]. Additionally, many typical antidepressants, such as paroxetine and imipramine, have been found to exert inhibitory effects on the expression of nNOS, the dominant synthase for NO production in the brain [17,18]. It is predicted that upcoming antidepressants may act partly through the NO signaling pathway and therefore could be useful for the treatment of drug-resistant depression [19,20]. Although it has been reported that the l-arginine-NO pathway may be involved in the antidepressant effects of ketamine in a rat model of forced swimming [21], the in-depth exploration of nNOS in ketamine has not yet been performed.

Hence, to explore the role of the nNOS signaling in the pathophysiology of depression and the mechanism underlying antidepressant action, we investigated the expression changes of nNOS, nNOS adaptor protein carboxy-terminal PDZ ligand of nNOS (CAPON), and the downstream physiological target protein Dexras1 in chronic unpredictable mild stress (CUMS) rats. In addition, our data also showed the effect of ketamine in depressive behavioral tests as well as the effect of ketamine on the expression of the above three proteins.

## 2 Materials and methods

### 2.1 Ethics and animals

Male adult Sprague-Dawley rats (210–260 g, 2–3 months old) were obtained from the Experimental Animal Center of Chongqing Medical University (Chongqing, China). They were housed one per cage with food and water available *ad libitum* and were maintained under standardized laboratory conditions (22 ± 2°C and 12 h light/12 h dark cycle, lights on at 7:00 a.m.) They acclimatized for 1 week before the experiments began. Rats were randomly divided into four groups (*n* = 12 in each group): a control group of healthy rats (group C), depression group (group D), control + ketamine group (group CK), and depression + ketamine group (group DK). Rats in groups D and DK were treated with CUMS. CUMS is a generally applicable animal model of depression used to produce an analog of depression in rats.


**Ethical approval:** The research related to animals’ use has been complied with all the relevant national regulations and institutional policies for the care and use of animals. The experimental protocols were performed in accordance with animal care guidelines of the NIH and the standards of the ethical care and use of laboratory animals issued by the State Committee of Science and Technology of China. These protocols were approved by the Ethical Committee of Chongqing Medical University. All efforts were made to minimize the suffering and the number of rats used in the current study.

### 2.2 Drugs and treatment schedule

**Figure 1:**
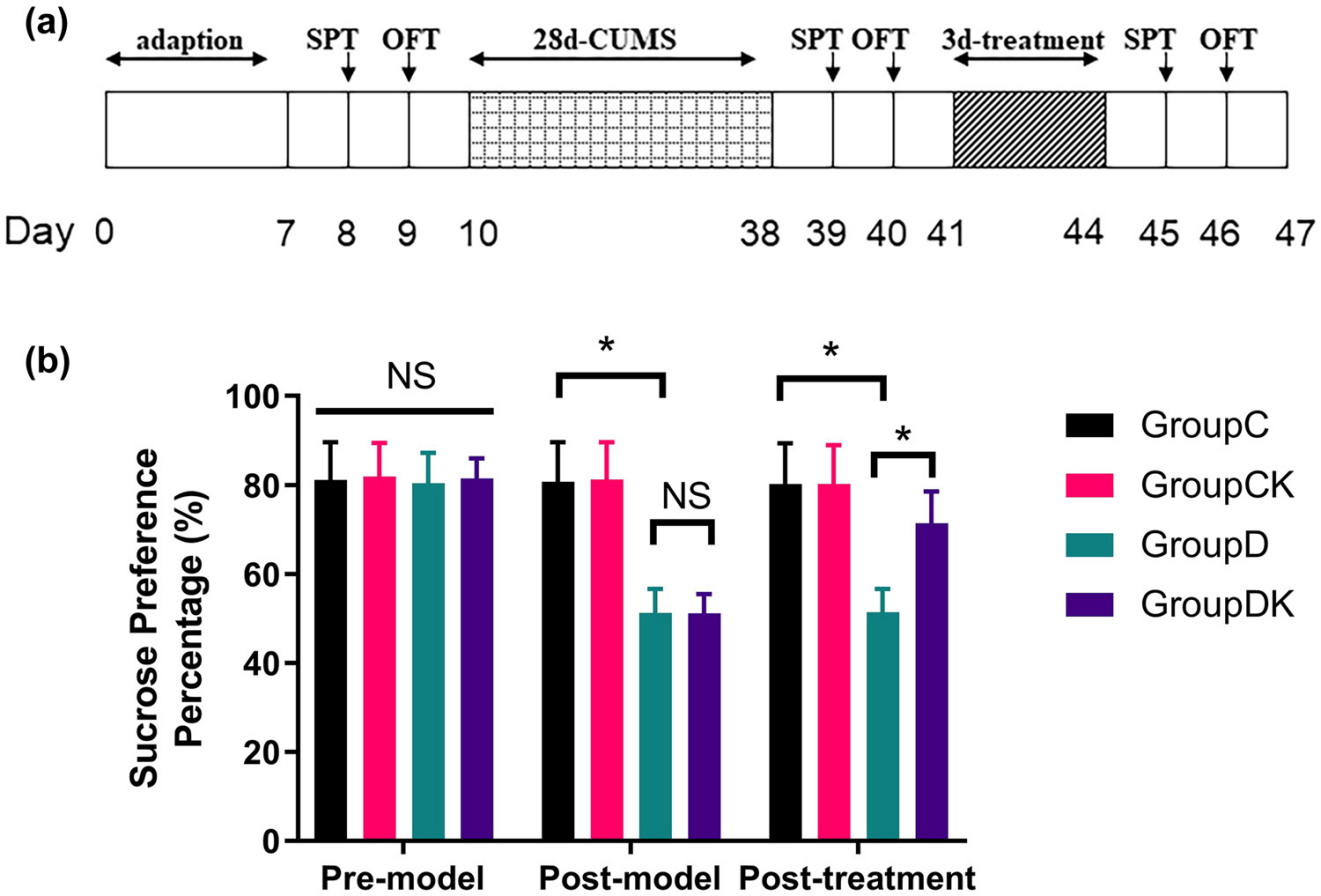
(a) Schematic overview of the experimental protocol. Adaption: the rats adapt for 1 week before further experiments, SPT: sucrose preference test, OFT: open field test, and CUMS: chronic unpredictable mild stress. (b) Effects of ketamine on behavioral performance of rats in the SPT. After CUMS procedure, baseline SPP of rats decreased in groups D and DK. SPP in group DK increased after ketamine treatment. Data of SPP (%) are presented as mean ± SD; ^*^ *P* < 0.05; NS, no significance; *n* = 12. Repeated measures analysis of variance was used, and the *P* value was corrected using Bonferroni’s method.

Ketamine (10 mg/kg, Sigma-Aldrich, MO, USA) was dissolved in saline (NaCl 0.9%) and freshly prepared every day before the intraperitoneal (IP) injections. Rats in groups C and D received an injection of 0.9% saline (IP), and rats in groups CK and DK received injection of 10 mg/kg ketamine (IP). All treatments were administered in a volume of 1 mL/kg. The aforementioned treatments were administered intraperitoneally once a day for 3 days. Dosing and treatment time for ketamine were selected based on previous studies [22–24], repeated ketamine administration to rats for 3 days (10 mg/kg; i.p.) caused significant antidepressant-like effects in CUMS model. The order of all the treatments (or sham treatments) and tests was as follows: 1. sucrose preference test (SPT) and open field test (OFT); 2. CUMS (groups C and CK were not exposed to this condition); 3. SPT and OFT; 4. IP administration of ketamine or saline according to the group assignment; 5. SPT and OFT; 6. immunohistochemistry, immunofluorescence, and real-time PCR analysis. All group assignments and treatments are shown in Figure 1a.

### 2.3 Stress-induced depression

A model of depression named CUMS was established as previously described [25,26]. The rats received no stresses (groups C and CK) or daily stressor stimuli (groups D and DK) for 28 days. Stressors consisted of the following conditions: food deprivation for 24 h, water deprivation for 24 h, continuous lighting for 24 h, housing in a soiled cage for 24 h, cage titling to 45° for 24 h, shaking for 15 min (1 shake/s), swimming in cold water 4°C for 5 min, swimming in hot water 45°C for 5 min, or tail pinching for 1 min. Briefly, one type of the stressor stimuli was applied randomly to the rats once daily between 9:00 a.m. and 12:00 p.m., except for the 24 h-duration stressors. The randomization was applied individually to each rat. The same stressor was not used on successive days to ensure unpredictability of the stimulation.

### 2.4 SPT

SPT was used to measure anhedonia in rodents. After water and food deprivation for 23 h, each rat was given free access to two identical bottles for 1 h; one bottle was filled with a 1% (wt/vol) sucrose solution and the other was filled with water. Each bottle was weighed before and after 1 h. The sucrose preference percentage (SPP) was calculated using the following formula: SPP (%) = (sucrose solution consumption (g)/(water consumption (g) + sucrose solution consumption (g)) × 100.

### 2.5 OFT

To evaluate spontaneous locomotor and exploratory activities of rats in a novel environment, OFT was adapted from the work of Luo et al. [27]. Each rat was placed at the center of a black square plexiglas arena (100 cm × 100 cm) surrounded by wall 50 cm high. Using a video computer tracking system (ZH-ZFT; Zhenghua Instruments, China), activity was recorded for 3 min. Total distance traveled (cm), the average velocity (cm/s), and the rearing counts (i.e., rearing counts sustained with only hind paws on the floor) were assessed for each rat. The floor was thoroughly cleaned by ethyl alcohol (70%) after each trial.

### 2.6 Tissue preparation

After OFT, rats were anesthetized with sodium pentobarbital (0.5%, 50 mg/kg, IP) and six rats in each groups were perfused transcardially with ice-cold phosphate-buffered saline ([PBS] pH 7.4) followed by 4% paraformaldehyde through the ascending aorta. The skull was subsequently opened to access the brain, which was used for histological studies. The other six rats were sacrificed under deep anesthesia, and the prefrontal cortex (PFC) was harvested for real-time PCR analysis.

For quantitative real-time PCR analysis, the PFC was carefully removed from the brain and dipped into 1 mL TRIzol (Life Technologies, USA), stored at −80℃ prior to RNA isolation. For histological studies, the prefrontal portion of the brain was blocked and post-fixed for 2 h in 4% paraformaldehyde. The specimens were transferred to a solution containing 30% sucrose in 0.1 M PBS overnight. The prefrontal portion of the brain containing the PFC was cut in 4 μm-thick sections by a rotary microtome (RM2135, Leica Instruments, Heidelberg, Germany).

### 2.7 Immunohistochemistry and immunofluorescence

After mounting on poly-l-lysine-coated slides, the 4 μm-thick sections were deparaffinized in dimethylbenzene, rehydrated successively in gradient ethanol, and washed with distilled water. Sections were washed with PBS and incubated in 3% hydrogen peroxide for 10 min at room temperature to block endogenous peroxidase activity. After antigen retrieval in a microwave for 20 min, the sections were incubated with 10% normal goat serum for 30 min at room temperature. The sections were incubated with nNOS antibody (1:50, Cell Signal Technology, Boston, MA, USA; 0.3% Triton X-100 in PBS) and CAPON (1:200, Santa Cruz Biotechnology, CA, USA) overnight at 4℃. Negative controls without primary antibodies were also included. After three washes with PBS, these sections were incubated with biotinylated secondary antibody (1:200; Zhongshan Golden Bridge Biological Company, Beijing, China) at room temperature for 30 min. The sections were incubated in avidin–biotin peroxidase complex for 30 min at room temperature; diaminobezidin was added as a chromogen and stained. Five randomly selected sections from each group were observed using an Olympus optical microscope (BX51, Olympus, Tokyo, Japan).

A double immunofluorescence labeling technique was applied to identify the expression of Dexras1. After antigen retrieval in a microwave for 20 min, the sections were incubated with 10% normal goat serum for 30 min at room temperature. The sections were incubated with Dexras1 antibody (1:50, Abcam, Cambridge, UK; 0.3% Triton X-100 in PBS) overnight at 4°C. Negative controls without primary antibodies were also included. These steps were performed in a dark place. After three washes with PBS, these sections were incubated with secondary antibody (1:200; FITC, Zhongshan Golden Bridge Biological Company, Beijing, China) at room temperature for 30 min. After washing in PBS three times for 5 min, the third antibody (1:10,000; PI, Sigma, MO, USA) was added for 1 min at room temperature. Sections were washed with PBS and wet-sealed in 50% glycerin. Five randomly selected sections from each group were observed using a fluorescence microscope (TCS-SP2, Leica Instruments, Heidelberg, Germany).

Image analyses were performed by a researcher blinded to the experimental conditions, and five fields from each section were imaged. The mean density of nNOS, CAPON, and Dexras1 in the PFC was analyzed using Image-Pro plus 6.0 image analysis software (Media Cybernetics, Bethesda, MD, USA).

### 2.8 Real-time PCR

Total RNA was isolated from the frozen specimens. The cDNA synthesis was carried out using the RevertAidTM RT Kit (Fermentas) in a 20 μL reaction volume according to the manufacturer’s protocol. The cDNA was diluted 1:1, and 2 μL was used in each 20 μL PCR reaction. The procedure for real-time PCR has been described previously [28]. The amplications were carried out in a 36-well plate in a 20 μL reaction volume containing 1× PCR buffer, 20 mM magnesium chloride, 0.2 mM deoxy NTP, 10 nmol TaqMan probe, 10 nmol of each forward (F) and reverse (R) primer, 1U of Taq polymerase, and 2 μL of plasmids or cDNA samples. Oligonucleotide primer pairs and probes for nNOS, CAPON, Dexras1, and β_2_-microglobin (β_2_-M) are shown in Table 1 in detail. Real-time PCR was performed in a Rotor-gene 3000 Detector (Corbett Research, CA, USA). The thermal profiles consisted of 3 min at 94℃ and 1 min at 60℃. All experiments were performed at least in triplicate for target gene and endogenous β_2_-M controls.

**Table 1:** Sequences of TaqMan probes and primers

Gene	Accession number	TaqMan probe (5′−3′)	Predicted size (bp)	Primers (5′−3′)
nNOS	NM 052799	ccttgttcacctcctccagcctgtcc	120	F-tccctctagccaaagaatttctcg
				R-ggtaggtgctggtgctttcaa
CAPON	NM 138922	cagccgagga taaccagccgat	144	F-gtgggcagccccttaggta
				R-gatgcctgactctcggaactt
Dexras1	NM 340809	tctggcaatc atccgtttcc cg	117	F-gcggcgaagt ctaccagttg
				R-tgtctaagct gaacaccagaatga
β2-M	NM 012512	cacccaccgagaccgatgtatatgcttgc	134	F-gtctttctacatcctggctcaca
				R-gacggttttgggctccttca

After computing the relative amounts of target gene and endogenous control for one sample, the final amount of target gene in that sample was presented as a ratio between the amount of target gene and amount of endogenous β_2_-M control. Relative mRNA of the control was normalized as 1.

### 2.9 Data analysis

All results are presented as mean ± deviation (SD). Statistical analysis was performed with SPSS version17.0 (SPSS Inc., Chicago, IL, USA) and GraphPad Prism 7.0 (GraphPad Prism Software). Statistical significance was determined using repeated measures analysis of variance repeatedly measured behavioral data, followed by Bonferroni’s *post-hoc* test for all the data. All other measures were analyzed using two-way analysis of variance at a single time point. Tukey’s multiple comparison test was used to compare the differences between the groups. *P*-values less than 0.05 were considered to be statistically significant.

## 3 Results

### 3.1 SPT

Results of SPT are shown in Figure 1b, there was no significant difference in their SPPs before depression model was established (*F* = 0.10, *P =* 0.962). After the CUMS procedure, rats in group D exhibited a decrease in their SPPs compared to rats in group C (*F* = 72.39, *P* < 0.001) and no significant difference was observed between groups D and DK. After the rats underwent 3 days intervention of ketamine or normal saline, the reduction in SPP in depression model was partially reversed by ketamine (*F* = 17.25, *P* < 0.001). In addition, ketamine had no significant effect on the control rats. These data indicate that ketamine can promote recovery of anhedonia after CUMS treatment.

### 3.2 OFT

**Figure 2:**
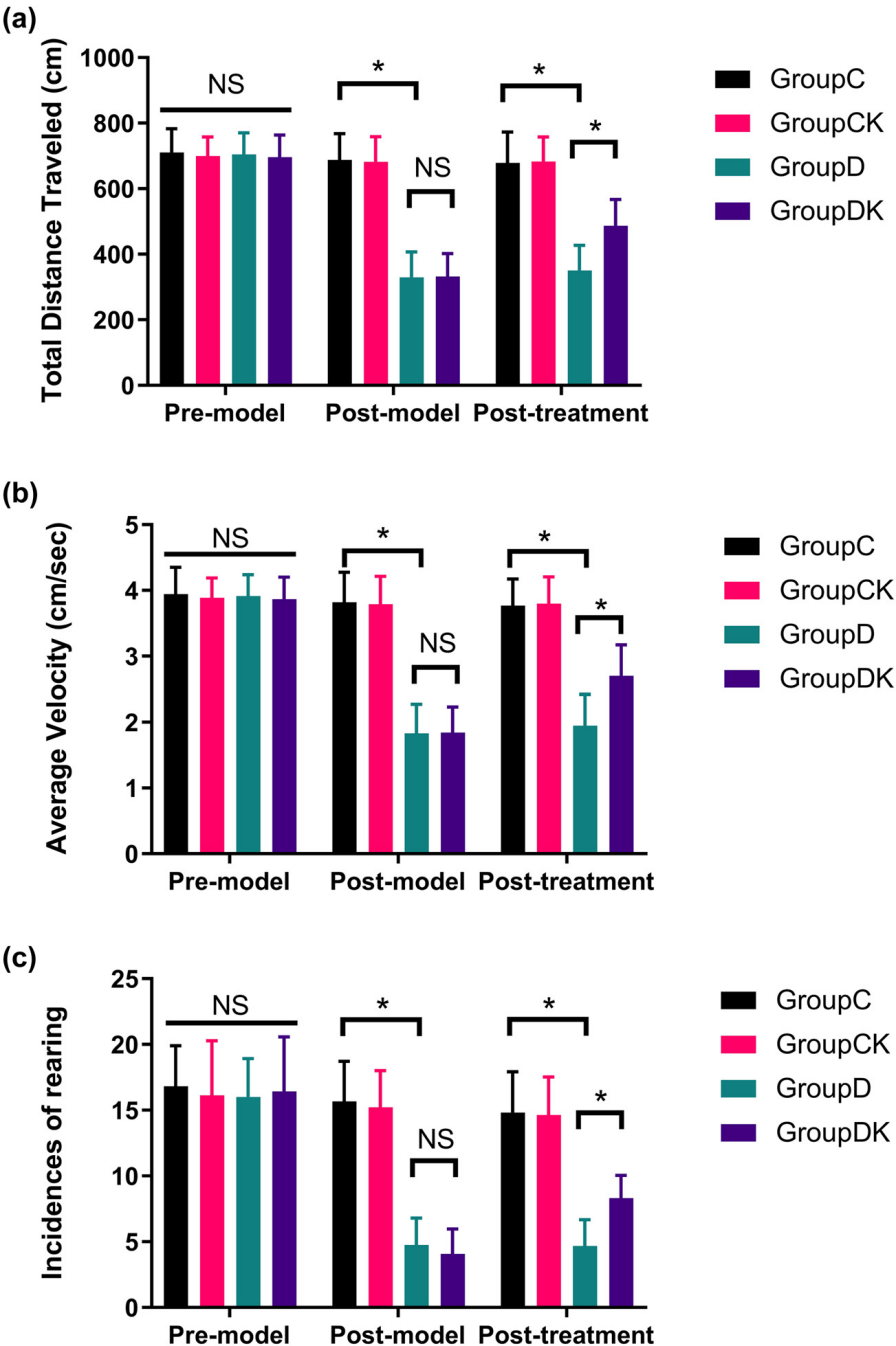

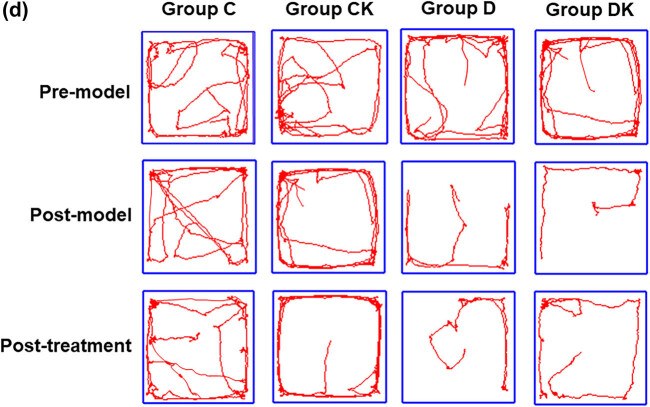
Effects of ketamine on the behavioral performance of rats in the OFT. After the CUMS procedure, the total distance traveled, the average velocity, and the rearing counts of the rats decreased in groups D. Ketamine reversed the decrease in the OFT parameters measured in CUMS rats: (a) total distance traveled, (b) average velocity, (c) rearing counts, and (d) representative photographs of rat motion trails in the square arena before and after modeling and treatment. Data of these scores are presented as mean ± SD; ^*^ *P* < 0.05; NS, no significance; *n* = 12. Repeated measures analysis of variance was used, and the *P* value was corrected using Bonferroni’s method.

Results of the total distance traveled, the average velocity, and rearing counts are shown in Figure 2a–c. Representative photographs of rat motion trails in the square arena before and after modeling and treatment are shown in Figure 2d. There was no significant difference among all groups in OFT scores before depression model was established (total distance traveled: *F* = 0.70, *P =* 0.556; average velocity: *F* = 0.70, *P =* 0.556; rearing counts: *F* = 0.15, *P* = 0.927). After the CUMS procedure, rats in group D exhibited a decrease in their OFT scores (total distance traveled: *F* = 86.38, *P* < 0.001; average velocity: *F* = 86.38, *P* < 0.001; rearing counts: *F* = 78.49, *P* < 0.001). After ketamine or normal saline treatment, ketamine partially restored depression-induced reduction of the OFT scores (total distance traveled: *F* = 16.56, *P* < 0.001; average velocity: *F* = 16.56, *P* < 0.001; rearing counts: *F* = 6.76, *P =* 0.013), while it did not affect the OFT scores in control group.

### 3.3 Immunohistochemistry and immunofluorescence

**Figure 3:**
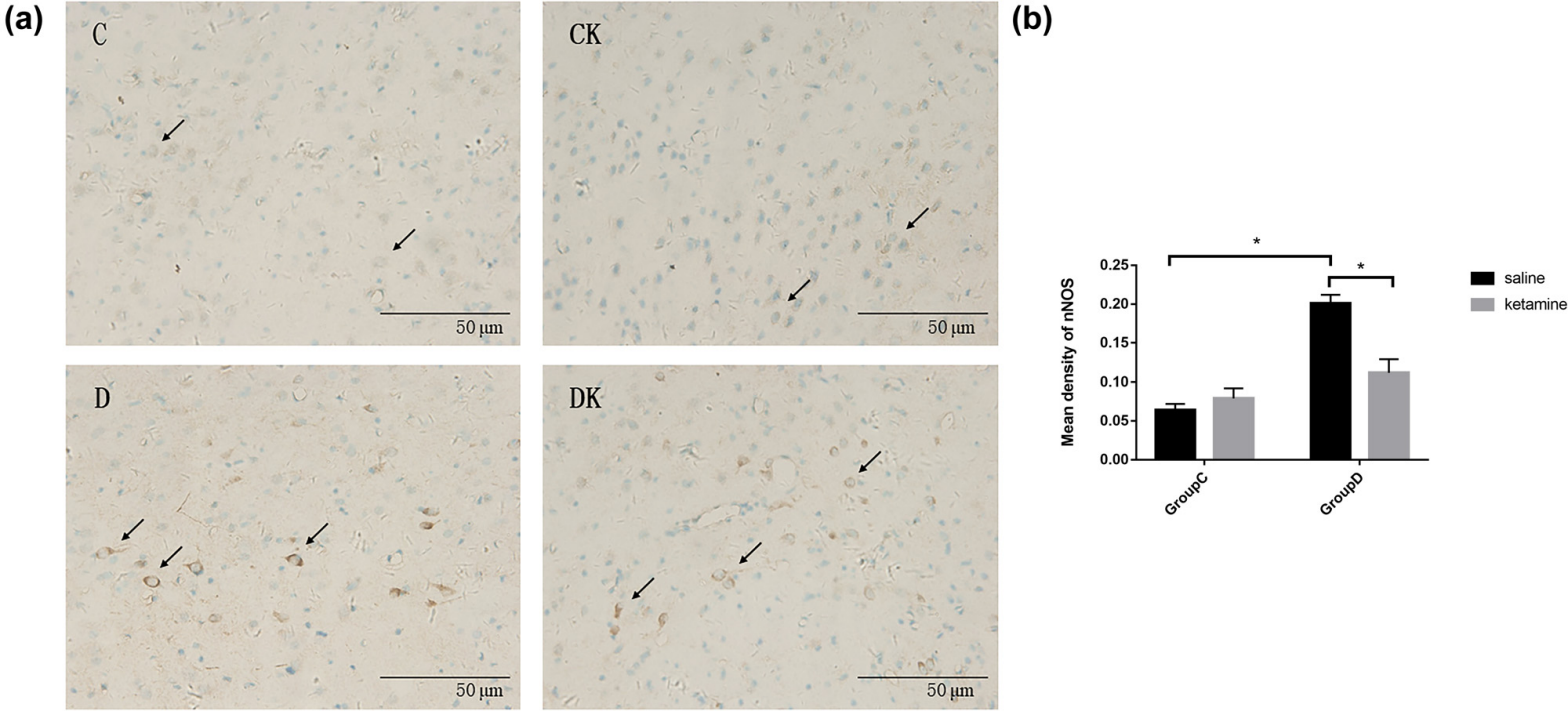
(a) Immunostaining using anti-nNOS antibody in the PFC. The prefrontal cortexes were obtained 24 h after the last behavioral test. Arrows indicate positive expression of nNOS. Scale bar = 50 μm. (b) Quantitative analyses of nNOS in the PFC. The mean density of nNOS in the PFC was increased by the CUMS procedure and decreased by ketamine treatment. Data of these scores are presented as mean ± SD; ^*^ *P* < 0.05; NS, no significance; *n* = 12. Tukey’s multiple comparison test was used to compare differences between the groups.

**Figure 4:**
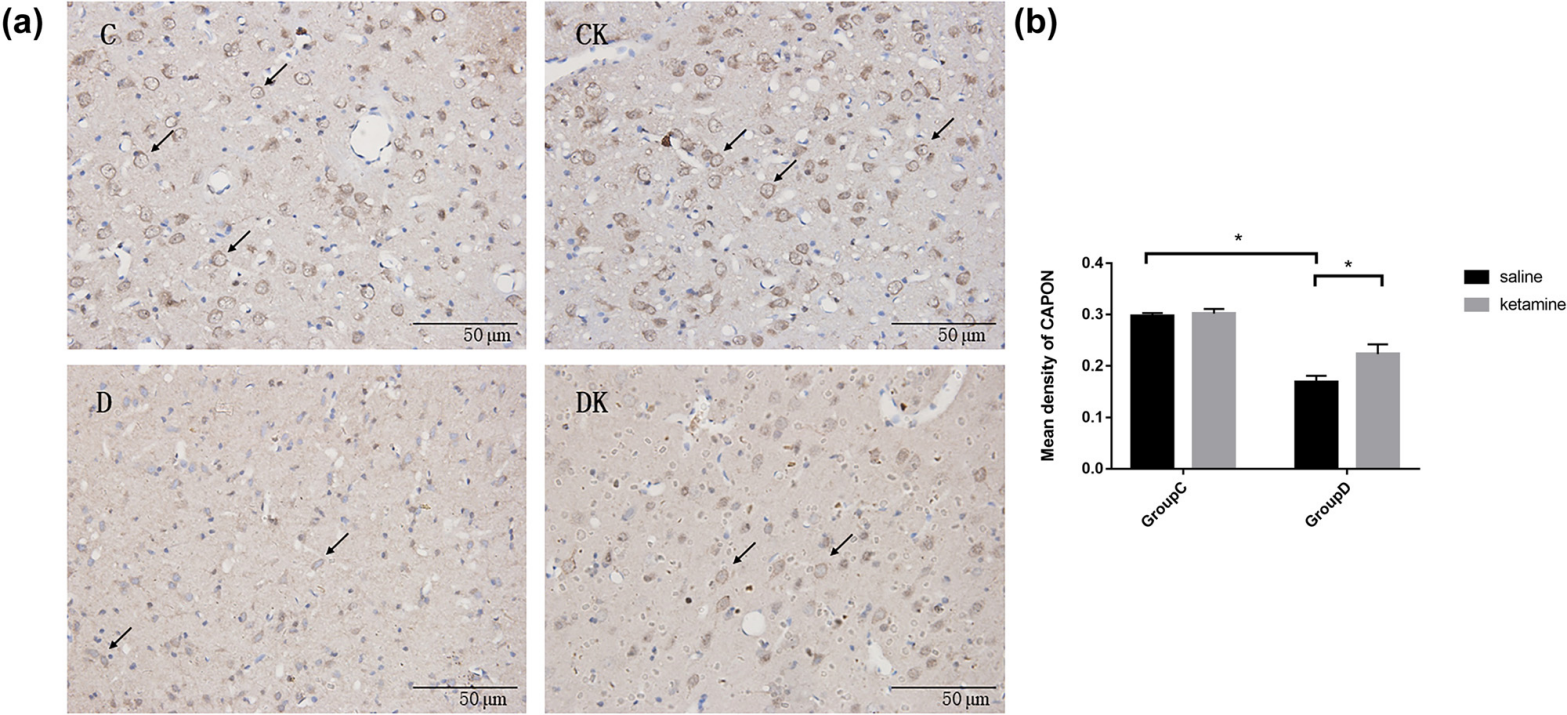
(a) Immunostaining using anti-CAPON antibody in the PFC. The prefrontal cortexes were obtained 24 h after the last behavioral tests. Arrows indicate positive expression of CAPON. Scale bar = 50 μm. (b) Quantitative analyses of CAPON in the PFC area. The mean density of CAPON in the PFC area were decreased by CUMS procedure and increased by ketamine treatment. Data of these scores are presented as mean ± SD; ^*^ *P* < 0.05; NS, no significance; *n* = 12. Tukey’s multiple comparison test was used to compare differences between the groups.

**Figure 5:**
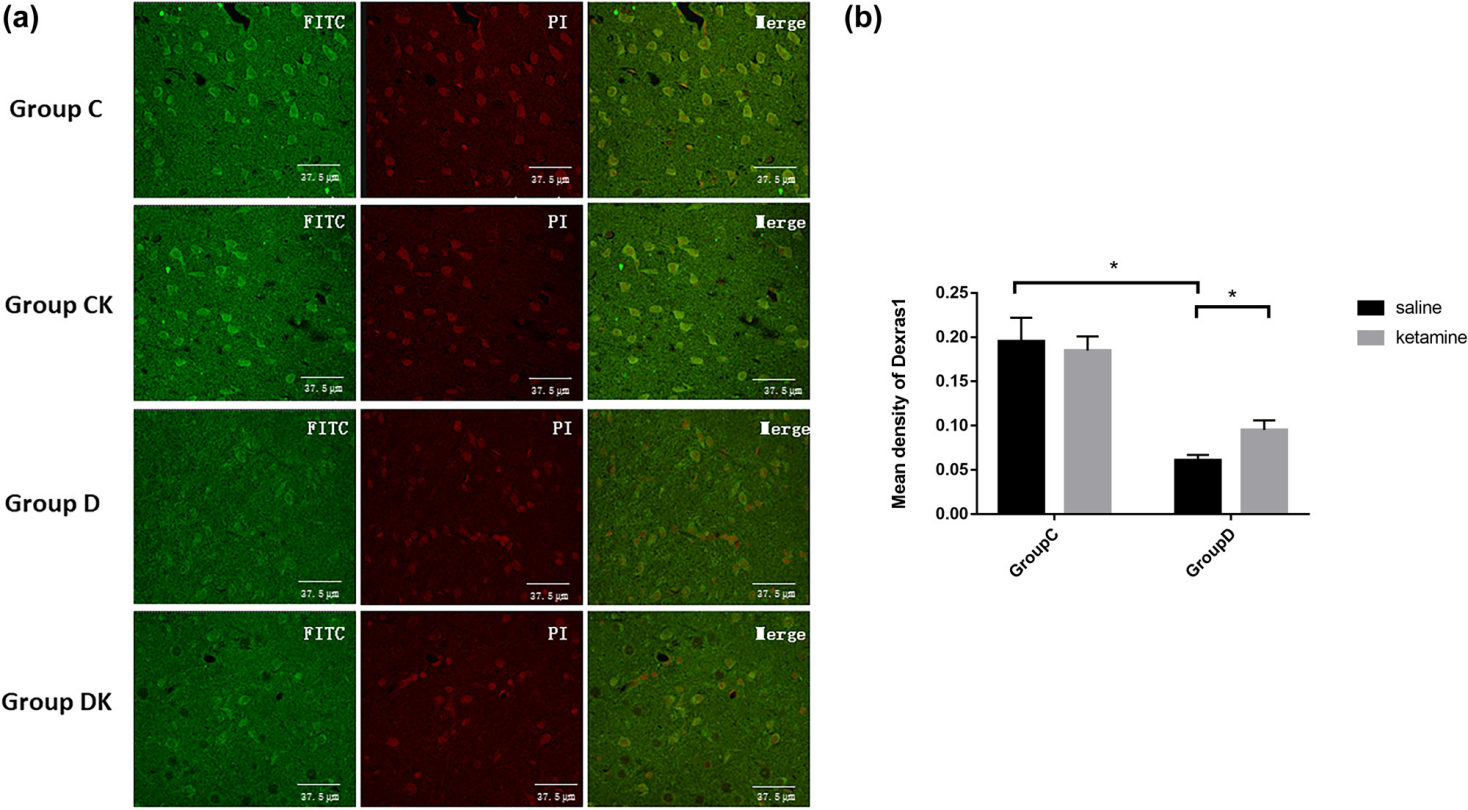
(a) Immunofluorescence using anti-Dexras1 antibody in the PFC. The prefrontal cortexes were obtained 24 h after the last behavioral test. Scale bar = 37.5 μm. (b) Quantitative analyses of Dexras1 in the PFC. The mean density of Dexras1 in the PFC was decreased by the CUMS procedure and increased by ketamine treatment. Data are presented as the mean ± SD; ^*^ *P* < 0.05; NS, no significance; *n* = 12. Tukey’s multiple comparison test was used to compare differences between the groups.

Levels of nNOS, CAPON, and Dexras1 expression in the PFC are shown in Figures 3–5 (Table S1). Compared with group C, a significant increase in the mean density of nNOS (*F* = 201.92, *P* < 0.001) and a decrease in the mean density of CAPON and Dexras1 (CAPON: *F* = 67.16, *P* < 0.001; Dexras1: *F* = 20.34, *P* < 0.001) in the PFC were observed in group D. Compared with group D, group DK showed reduced expression of nNOS (*F* = 102.23, *P* < 0.001) and enhanced expression of CAPON and Dexras1 (CAPON: *F* = 46.32, *P* < 0.001 and Dexras1: *F* = 6.05, *P =* 0.017) in the PFC.

### 3.4 Real-time PCR

**Figure 6:**
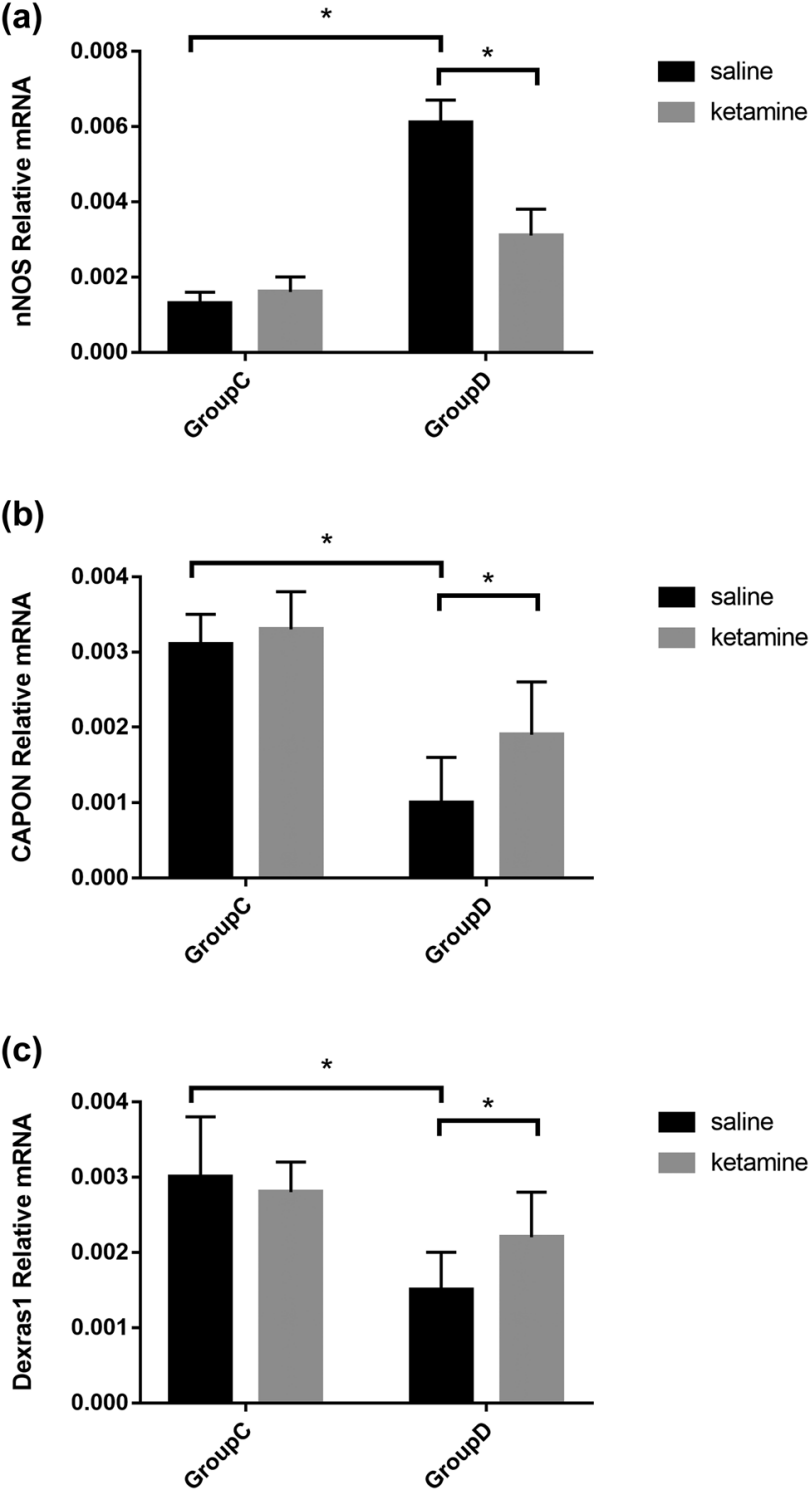
Analysis on mRNA expression for nNOS, CAPON, and Dexras1 in PFC of the rat by TaqMan quantitative real-time PCR. (a) nNOS mRNA expression in the PFC was increased by the CUMS procedure and decreased by ketamine treatment. (b) CAPON mRNA expression in the PFC was decreased by the CUMS procedure and increased by ketamine treatment. (c) Dexras1 mRNA expression in the PFC was increased by the CUMS procedure and decreased by ketamine treatment. Relative mRNA of the samples was normalized to that of β_2_-M. Data of these scores are presented as mean ± SD; ^*^ *P* < 0.05; NS, no significance; *n* = 12. Tukey’s multiple comparison test was used to compare differences between the groups.

Gene expression patterns for nNOS, CAPON, and Dexras1 in the PFC are shown in Figure 6 (Table S2). Compared with group C, a significant increase in the mRNA of nNOS (*F* = 118.82, *P* < 0.001) and a decrease in the mRNA of CAPON and Dexras1 (CAPON: *F* = 116.72, *P* < 0.001; Dexras1: *F* = 37.53, *P* < 0.001) in the PFC were observed in group D. Compared with group D, the reduced expression of nNOS (*F* = 79.53, *P* < 0.001) and enhanced expression of CAPON and Dexras1 (CAPON: *F* = 11.52, *P =* 0.002 and Dexras1: *F* = 6.89, *P =* 0.012) in the PFC were significantly attenuated by ketamine.

## 4 Discussion

In this study, we provide evidence that administration of ketamine for 3 days induced antidepressant-like effects in CUMS rats. Moreover, it was demonstrated that 3 days of ketamine treatment mitigated the depression-induced excessive expression of nNOS and up-regulated the expression of CAPON and Dexras1, and restored the efficacy balance between total nNOS and the nNOS–CAPON–Dexras1 complex. Furthermore, our data suggest that ketamine may not affect depressive behavior and nNOS-related protein changes in the control group.

CUMS was applied to establish the animal model of depression and is considered one of the most valid animal depression models. CUMS application in rat models can induce anhedonia, the core symptom of depression, which can be reversed by antidepressants [29]. The SPT was applied to evaluate the rats’ levels of anhedonia, which was reflected by the reduction of the SPP [4,7]. We found that the rats exposed to CUMS displayed a significant decrease in the SPP, while the reduction in their SPP could be mitigated by 3 days of administration of small doses of ketamine. In addition, ketamine did not seem to affect depression-like states in the control group. This outcome revealed that the model of depression was successfully reproduced in these rats.

The injection regimen of 10 mg/kg ketamine (IP) for 3 consecutive days was chosen based on previous studies [23,24]. Ketamine shows a significant advantage over typical antidepressant drugs because of its rapid efficacy [30,31]. For the time of administration, we chose 3 days for two reasons. One was that while single ketamine administration consistently elicits antidepressant effects, the results of studies employing repeated administration of ketamine on CUMS are less conclusive [32]. Furthermore, typical antidepressant drugs take about a week to elicit antidepressant effects. In clinical practice, therapy for depression requires the chronic administration of antidepressant drugs. A large number of functional imaging, lesion, and brain stimulation studies have indicated that the PFC is essential for depression [33]. Previous studies have reported the effects of ketamine on the PFC in animal models of depression [34–36]. To highlight the pharmacodynamic characteristics of ketamine and to simulate clinical practice, this protocol of ketamine administration was applied in the present study to observe the changes in the PFC.

There are many hypotheses about the pathogenesis of depression, but the biological mechanisms remain unclear. It has been generally accepted to be associated with glutamate neurotransmission, as reduced levels of glutamate in the PFC from depressed patients were reported [37]. While in the brains from the Stanley Consortium, increased nNOS immunoreactivity has been reported in patients with depression and bipolar disorder [38]. Consistent with this, the depressive rats showed a higher level of expression of nNOS in the PFC in our results. Besides, we evaluated the expression of nNOS adaptor protein CAPON and CAPON downstream binding protein Dexras1, a member of the ras protein, which interacted with nNOS via CAPON [39,40]. Three proteins nNOS, CAPON, and Dexras1 interact to form a complex. In our study, contrary to nNOS expression, the expression of CAPON and Dexras1 was lower in the CUMS rats, which was up-regulated by ketamine administration. nNOS is closely connected to NMDA receptors, once NMDA receptors are activated, the influx of Ca^2+^ and activated CaM bind and activate nNOS to produce NO [41,42], which is widely proposed to be involved in the pathogenesis of depression. NO signaling may be involved in the antidepressant-like effects of MK-801 (Dizocilpine), the NMDA receptor antagonist, as previous study found in the mouse forced-swim test [43].

The suppression of excessive nNOS expression by ketamine may be partly through up-regulation of CAPON expression. Jiang et al. [44] demonstrated that NO donor treatment enhanced CAPON expression in astrocytes, and the increased CAPON bound to nNOS to negatively regulate its activity partly by facilitating CAPON–nNOS nuclear localization. CAPON plays an important role in a feedback mechanism in the context of the modulation of nNOS activity. In the PFC of the CUMS rats, nNOS expression was elevated. Regarding the feedback mechanism, CAPON expression would subsequently be expected to be elevated. However, the outcomes showed a reduction of CAPON expression in the depressed subjects. This disproportionate change in expression level would lead to an imbalance of function, which may be involved in the pathogenesis of depression. Sub-anaesthetic doses of ketamine administration suppressed the elevated expression of nNOS and up-regulated the expression of CAPON to restore the balance between nNOS and CAPON at both the expression and functional levels. Consistent with changes in CAPON expression, Dexras1 expression within the PFC was reduced in CUMS rats. This reduction was partly reversed by 3 days of ketamine administration. The effect of ketamine of up-regulating Dexras1 expression may help to restore the efficacy balance between total nNOS and the nNOS–CAPON–Dexras1 complex.

In the present study, we used a rat model of depression as well as normal control rats to investigate the expression changes in nNOS, CAPON, and Dexras1 and the effect of 3 days of ketamine administration on their expression in the PFC. As the results showed, significant changes in nNOS, CAPON, and Dexras1 expression were found between the different groups of rats, and 3 days of ketamine administration influenced the expression of these three proteins. Due to the limitations of the study, a more detailed elucidation of the role of the nNOS–CAPON–Dexras1 complex in the pathogenesis of depression and antidepressant effects of ketamine will need further study. It has been predicted that upcoming antidepressants may act partly through the NO signaling pathway and therefore could lead to the development of a novel class of treatment interventions for depression.

## 5 Conclusion

In conclusion, three consecutive days of ketamine administration significantly improved depressive behavior in rats. Additionally, alterations in the expression of nNOS, CAPON, and Dexras1 may be involved in the pathogenesis of depression and the antidepressant-like effects of ketamine. nNOS–CAPON–Dexras1 complex may be a novel target for the treatment of depression and the antidepressant effect of ketamine.

## Supplementary Material

Supplementary Table
