# Supplementary material for: Ketamine alleviating depressive-like behaviors is associated with regulation of nNOS–CAPON–Dexras1 complex in chronic unpredictable mild stress rats
Source: Transl Neurosci. 2022 Sep 21;13(1):309–19. doi: 10.1515/tnsci-2022-0245 (PMC9508647; doi:10.1515/tnsci-2022-0245)
Supplement: Supplementary Table [file tnsci-2022-0245-sm.pdf]

Supplementary material

Table S1: Levels of nNOS, CAPON and Dexras1 expression in the prefrontal cortex by Immunohistochemistry and immunofluorescence (n = 12)

Groups	nNOS	CAPON	Dexras1
C	0.79 ± 0.02	0.10 ± 0.03	0.25 ± 0.02
CK	0.65 ± 0.03$^{\#}$	0.17 ± 0.04$^{\#}$	0.31 ± 0.02$^{\#}$
D	0.78 ± 0.02	0.09 ± 0.01	0.25 ± 0.01
DK	0.05 ± 0.01$^{\blacktriangle\square}$	0.28 ± 0.03$^{\blacktriangle\square}$	0.46 ± 0.02$^{\blacktriangle\square}$

Compared with group D3, $^{\#}P < 0.05$; Compared with group D7, $^{\blacktriangle}P < 0.05$; Compared with group F7, $^{\square}P < 0.05$.

Data were shown as mean value ± SD, Tukey's multiple comparison test used to compare differences between the groups.

Table S2: Expression of nNOS, CAPON and Dexras1 mRNA in prefrontal cortex of rats in each group (n = 12)

Groups	nNOS mRNA	CAPON mRNA	Dexras1 mRNA
C	0.0013 ± 0.0003	0.0031 ± 0.0004	0.0030 ± 0.0008
CK	0.0016 ± 0.0004	0.0033 ± 0.0005	0.0028 ± 0.0004
D	0.0061 ± 0.0006$^{\#}$	0.0010 ± 0.0006$^{\#}$	0.0015 ± 0.0005$^{\#}$
DK	0.0031 ± 0.0007$^{\blacktriangle}$	0.0019 ± 0.0007$^{\blacktriangle}$	0.0022 ± 0.0006$^{\blacktriangle}$

Compared with group C, $^{\#}P < 0.05$; Compared with group D, $^{\blacktriangle}P < 0.05$.

Data were shown as mean value ± SD, Tukey's multiple comparison test used to compare differences between the groups.
